# MicroRNA‐130a‐3p Targets the Androgen‐Related Transcription Factor *MAFB*: Effects on Proliferation, Migration, Apoptosis, and Cell Cycle in Hypospadias

**DOI:** 10.1002/pdi3.70062

**Published:** 2026-06-30

**Authors:** Jiaxin Zhou, Yixuan Wang, Zhicheng Zhang, Qiang Zhang, Zhenmin Liu, Hongsong Chen, Xingguo Luo, Chunlan Long, Lianju Shen, Xing Liu, Guanghui Wei

**Affiliations:** ^1^ Department of Urology Children's Hospital of Chongqing Medical University National Clinical Research Center for Children and Adolescents' Health and Diseases Ministry of Education Key Laboratory of Child Development and Disorders Chongqing China; ^2^ Chongqing Key Laboratory of Structural Birth Defect and Reconstruction Chongqing China

**Keywords:** cell apoptosis, cell cycle, hypospadias, *MAFB*, miR‐130a‐3p

## Abstract

Hypospadias is among the most frequent congenital malformations of the male genitalia. The expression of *V‐maf musculoaponeurotic fibrosarcoma oncogene homolog B* (*MAFB*), a transcription factor within the androgen signaling pathway, is closely correlated with the etiology of this condition. From an epigenetic perspective, this study investigated the role of microRNA‐130a‐3p (miR‐130a‐3p) in regulating *MAFB* during hypospadias pathogenesis. Clinical foreskin samples from hypospadias children and the dioctyl phthalate (DEHP)‐induced mouse genital tubercle tissues were analyzed. The expression levels of miR‐130a‐3p and *MAFB* were detected by qRT‐PCR and western blot. In vitro, miR‐130a‐3p was modulated in HS68 cells; CCK‐8, scratch assay, western blot, flow cytometry, and dual‐luciferase assay were used to assess its effects and targeting of *MAFB*. Our study demonstrated that miR‐130a‐3p targeted and suppressed *MAFB* expression. This suppression inhibited cellular proliferation and migration and reduced *Vimentin* mRNA expression. Cell cycle distribution was disrupted, marked by Gap 1 phase increase and Synthesis phase reduction, and the cell apoptosis rate increased. Cyclin‐dependent kinase 2 (CDK2), Cyclin E1, and Proliferating Cell Nuclear Antigen (PCNA) were downregulated. miR‐130a‐3p targets and inhibits *MAFB* expression, disrupting the normal processes of cell proliferation, migration, cell apoptosis, and cell cycle progression, ultimately leading to the development of hypospadias.

## Graphical abstract

MicroRNA‐130a‐3p targets and inhibits *MAFB* expression, leading to the development of hypospadimas. MicroRNA‐130a‐3p inhibits the expression of *MAFB*, disrupts the normal processes of cell proliferation, migration, cell apoptosis, and cycle progression, and ultimately leads to the development of hypospadias.

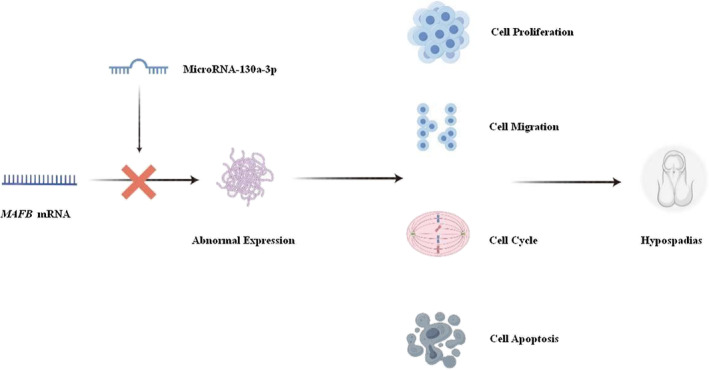

## 1 Introduction

Hypospadias is the most common congenital structural anomaly of the male urethra [1]. This urogenital malformation originates during embryonic development, typically between weeks 7 and 14 of gestation. It results from impaired fusion of the urethral folds [2, 3]. Current evidence indicates that hypospadias arises from multifactorial interactions involving genetic, endocrine, and environmental elements.

Genomic studies have identified several mutations associated with hypospadias, including genes such as *Wilms Tumor 1*, *Steroidogenic Factor 1*, *Sex‐determining Region Y*, and *Steroid 5 Alpha‐Reductase 2* [4, 5, 6]. These findings highlight the genetic complexity of this disorder.

*MAFB* is a basic leucine zipper transcription factor. It plays critical roles in organ development and cell differentiation [7]. Specifically, *MAFB* is crucial for the development of the male reproductive organs. A study by Suzuki et al. revealed that *Mafb* is highly expressed in interstitial cells of the mouse urogenital sinus (UGS). Conditional knockout of *Mafb* in these cells led to hypospadias in mice. Mechanistically, MAFB acts upstream of the androgen receptor (AR) pathway. It binds directly to the *AR* promoter, enhancing its transcription and ensuring proper urethral formation [8].

MicroRNAs (MiRNAs), which are 18–24 nucleotides long, belong to small non‐coding RNAs. They control gene expression during the post‐transcriptional stage by combining with the 3′‐untranslated region (3′‐UTR) segment of the target mRNAs, leading to translational suppression or mRNA degradation [9, 10, 11, 12]. MiRNAs control many biological processes, including cell cycle and cell apoptosis. Previous studies indicate that microRNA‐130a‐3p (miR‐130a‐3p) can promote cancer cell proliferation and invasion by targeting hormone receptors [13]. This suggests a potential role in hormone‐mediated development.

Our prior clinical research showed reduced *MAFB* expression in foreskin tissues from hypospadias patients [14]. However, whether miR‐130a‐3p regulates *MAFB* remains unclear. Bioinformatics analysis using TargetScan predicted a combination site for the miR‐130a‐3p with the 3′‐UTR of *MAFB* mRNA.

Therefore, this study aims to clarify how miR‐130a‐3p regulates *MAFB*. Using in vitro models, we explore it by cell proliferation, migration, cell apoptosis, and cell cycle progression in HS68 cells, providing new perspectives into the molecular mechanisms of hypospadias.

## 2 Materials and Methods

### 2.1 Clinical Specimen Collection

Foreskin samples were randomly collected from 15 children aged 1–7 years with hypospadias and 15 children aged 1–7 years with phimosis undergoing circumcision at the Children's Hospital of Chongqing Medical University from August 2024 to October 2024. No cases of undescended testicles, intersex conditions, or endocrine anomalies were detected among the hypospadias children. Children had not been treated with hormones or immunosuppressive drugs. All samples were obtained under informed consent.

### 2.2 Construction of an Animal Model of Hypospadias

Sixteen female and eight male clean and healthy eight‐week‐old C57BL/6J mice were purchased from the Experimental Animal Center of Chongqing Medical University. Feed and drinking water were freely available, with a humidity of 50%–60% and a temperature of 22°C–26°C. After 5 days of rearing, the C57BL/6J mice were weighed. Female and male mice were mated at a ratio of 2:1. Mice were caged together overnight and separated the next morning. After 10 days, they were weighed again. Based on the initial weight and whether the abdomen was protruding, it was determined whether the female mice were successfully pregnant. The day of separation was counted as the gestational day 0 (GD 0) of pregnancy for the successfully pregnant female mice. Starting from the GD 11.5, the mice were randomly divided into two groups, separately gavaged with 500 mg/kg DEHP or corn oil at the same time for 7 consecutive days. After the pregnant female mice gave birth to newborn mice, the genital tubercle (GT) tissues of the newborn mice were taken and sent for scanning electron microscopy (SEM).

### 2.3 Cell Transfection

miR‐130a‐3p inhibitor, miR‐130a‐3p mimics, negative control (NC) inhibitor, NC mimics, and pcDNA3.1‐*MAFB* (*MAFB*‐overexpressing plasmid) were purchased from Beijing Tsingke Biological Technology Co. Ltd. Lipofectamine 2000 reagent was added to Opti‐MEM reduced‐serum medium. Then, functional small RNAs and plasmids were added to a separate aliquot of Opti‐MEM reduced‐serum medium. After 5 minutes, the two solutions were mixed, kept for 20 minutes, and then added to the cell culture dishes, and the dishes were gently swirled to ensure uniform mixing. After 6 hours, transfection was terminated.

### 2.4 qRT‐PCR Detection

qRT‐PCR was performed after extracting total RNA, performing reverse transcription, and analyzing gene expression. The primers used in this study were purchased from Tsingke. Reverse primers of miR‐130a‐3p and U6 were miRNA Universal Adaptor PCR Primers (QP029) purchased from GeneCopoeia. The other primer sequences are presented in Table 1.

**TABLE 1:** Primer sequences used in quantitative RT‐PCR.

Primers	Sequence (5′→3′)	
β‐actin	Forward	CCTGGCACCCAGCACAAT
	Reverse	GGGCCGGACTCGTCATAC
*α‐SMA*	Forward	CTGCTGAGCGTGAGATTGTC
	Reverse	CTCAAGGGAGGATGAGGATG
*Vimentin*	Forward	CAGCTGCACCTGACGCCCTT
	Reverse	GTATCAACCAGAGGGAGTGA
MAFB/Mafb	Forward	GACGCAGCTCATTCAGCAG
	Reverse	CTCGCACTTGACCTTGTAGGC
U6	Forward	ATGGACTATCATATGCTTACCGTA
miR‐130a‐3p	Forward	CAGCAGTGCAATGTTAAAAGG

### 2.5 Western Blot Analysis

Protein extraction, membrane transfer, and membrane blocking were performed sequentially. Primary antibodies and secondary antibodies were added to the membranes. Equal volumes of chemiluminescent reagent Solution A and Solution B were mixed, and the chemiluminescence was detected using a chemiluminescence analyzer. Subsequently, protein expression levels were analyzed.

### 2.6 Evaluation of Cell Viability

The CCK‐8 assay assessed cell viability. After seeding cells into plates for 1 day, cell transfection was performed. At 0, 24, 48, and 72 h post‐transfection, the complete medium containing CCK‐8 working solution was added to the cells, followed by a 2‐h incubation. The absorbance at 450 nm was measured, and the cell proliferation rate was analyzed.

### 2.7 Cell Scratch Assay

When the transfected cells in the 6‐well plates reached nearly full confluence, we made 3 scratches per experimental group using 10 μL pipette tips and captured images at 0 and 24 h to analyze the healing rate. Healing Rate (%) = (initial scratch area − residual scratch area)/(initial scratch area) × 100%.

### 2.8 Flow Cytometric Analysis of the Cell Cycle and Apoptosis

At 48 h after cell transfection, cells in the culture dishes were digested into single cells using 0.25% trypsin. The cell pellet was resuspended in 100 μL PBS, and 900 μL pre‐chilled 75% ethanol was slowly added with gentle vortexing, followed by overnight fixation at 4°C. After centrifugation and resuspension, we kept the cells at room temperature for 15 min. Following another centrifugation, RNase A was added for resuspension, and the cells were incubated in a 37°C water bath for 30 min. Then we added PI staining solution, mixed well, incubated at 4°C in the dark for 30 min, and detected the cell cycle distribution. For apoptosis analysis, we resuspended the cell pellet in 1 × Annexin V binding buffer, added Annexin V‐FITC and PI staining solution, mixed gently by vortexing, incubated at room temperature in the dark for 20 min, and detected cell apoptosis by flow cytometry.

### 2.9 Dual‐Luciferase Reporter Gene Assay

**FIGURE 1:**
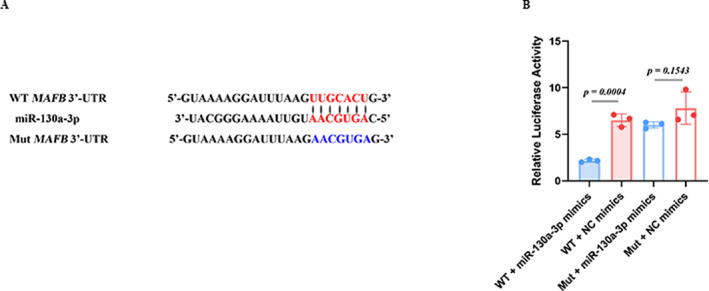
Construction of pmirGLO plasmids and dual‐luciferase reporter assay results. (A) Sequences of WT‐*MAFB* 3′UTR and Mut‐*MAFB* 3′UTR. (B) Verification of microRNA‐130a‐3p targeting Mafb via dual‐luciferase reporter assay. 3′‐UTR, 3′‐untranslated region; Mut, mutant; WT, wild‐type.

We cloned wild‐type (WT) and mutant (Mut) 3′‐UTR sequences of Mafb into pmirGLO vectors (Figure 1A). These were co‐transfected with functional small RNAs into HEK‐293T cells. After 48 hours, luciferase activity was measured.

### 2.10 Statistical Analysis

Data are presented as mean ± standard deviation (SD). Comparisons used Student's *t*‐test, Mann–Whitney *U* test, or ANOVA. *p* < 0.05 was considered significant. All analyses were performed with GraphPad Prism 10.0.

## 3 Results

### 3.1 Upregulation of miR‐130a‐3p and Downregulation of *MAFB* in Hypospadias Tissues

**FIGURE 2:**
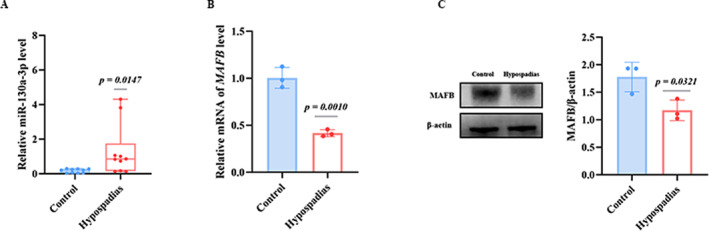
Expression of microRNA‐130a‐3p and *MAFB* in clinical specimens. (A–C) Quantitative RT‐PCR and western blot were used to detect the expression of microRNA‐130a‐3p and *MAFB* in foreskin tissues of children with hypospadias and children undergoing circumcision.

Fifteen control samples from children aged 1–7 years (5.1 ± 1.7 years) and 15 hypospadias samples obtained from children aged 1–7 years (4.2 ± 1.8 years), with no difference in the age of children (*p* = 0.17), were selected. Compared with the control group, clinical foreskin samples of children with hypospadias demonstrated an elevation in miR‐130a‐3p expression (Figure 2A), whereas *MAFB* levels were reduced in these tissues (Figure 2B–C).

### 3.2 Upregulation of miR‐130a‐3p and Downregulation of *Mafb* in the DEHP‐Induced Mouse Model

**FIGURE 3:**
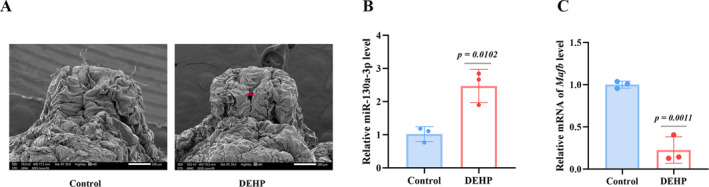
Establishment of DEHP‐induced hypospadias mouse models and expression of microRNA‐130a‐3p and *Mafb* in GT tissues. (A) SEM images of GT tissue in the DEHP‐induced hypospadias mouse models. (B–C) qRT‐PCR was used to detect the expression of microRNA‐130a‐3p and *Mafb* in the GT tissues from DEHP‐induced hypospadias mice and normal control mice.

Ten C57BL/6J female mice were confirmed pregnant. All five pregnant mice in the DEHP group delivered 6.60 ± 0.55 pups per litter. Of the 3.40 ± 1.14 male pups per litter in the DEHP group, 2.20 ± 0.84 mice exhibited hypospadias. All five mice in the corn oil group delivered 6.40 ± 1.14 pups per litter, including 3.80 ± 1.10 male pups per litter. A DEHP‐induced hypospadias mouse model was successfully established in this study. SEM discovered that the urethral meatus was located ventrally on the penis (Figure 3A). Subsequent detection of GT tissues from hypospadias pups demonstrated a significant elevation in miR‐130a‐3p expression compared with the normal pups (Figure 3B), whereas *Mafb* expression was notably suppressed in the same tissue samples (Figure 3C).

### 3.3 *MAFB* Silencing Suppresses HS68 Cell Functions

**FIGURE 4:**
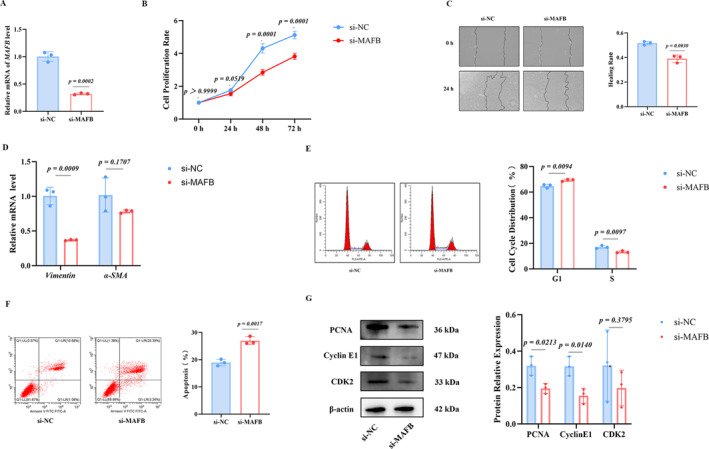
Inhibition of *MAFB* expression and detection of cell proliferation, cell migration, cell apoptosis, and cell cycle‐related changes in HS68 cells. (A) Quantitative RT‐PCR detection of *MAFB*. (B) CCK‐8 was used to detect cell proliferation at 0, 24, 48, and 72 h after transfection. (C) The cell migration ability was assessed by the scratch method. (D) Quantitative qRT‐PCR detection of *Vimenti* *n* and *α‐SMA*. (E) Cell cycle distribution was analyzed by flow cytometry. (F) Cell apoptosis was analyzed by flow cytometry. (G) Western blot was used to detect the expression of CDK2, Cyclin E1, and PCNA. *α‐SMA*, *α‑smooth muscle actin*; CDK2, Cyclin‐dependent kinase 2; PCNA, Proliferating Cell Nuclear Antigen.

In comparison with the silent negative control (si‐NC) group, the results were as follows. Transfection of si‐MAFB significantly reduced the expression level of *MAFB* mRNA to 0.3‐fold of the control level (Figure 4A). On day 2 and day 3 after transfection, the cell proliferation ability decreased significantly (Figure 4B), and the cell migration ability was significantly inhibited (Figure 4C); *Vimentin* level decreased, whereas *α* *‐smooth muscle actin* (*α* *‐SMA*) level showed no significant difference (Figure 4D). Flow cytometry analysis results revealed that the percentage of the gap 1 (G1) stage increased and the percentage of the synthesis (S) stage decreased (Figure 4E), apoptosis rate increased (Figure 4F), the expressions of CyclinE1 and PCNA were significantly downregulated, whereas expression of protein CDK2 showed no significant decrease (Figure 4G).

### 3.4 Inhibiting miR‐130a‐3p Enhances *MAFB* and Improves Cell Function

**FIGURE 5:**
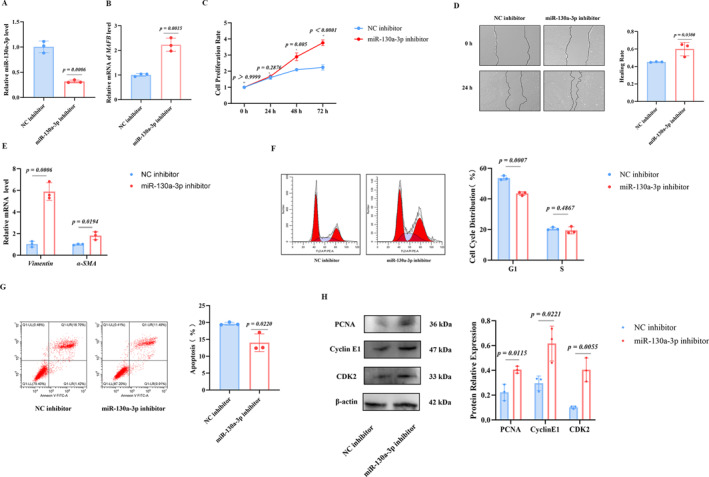
Inhibition of microRNA‐130a‐3p expression and detection of cell proliferation, cell migration, cell apoptosis, and cell cycle‐related changes in HS68 cells. (A–B) Quantitative RT‐PCR detection of microRNA‐130a‐3p and *MAFB* mRNA. (C) CCK‐8 was used to detect cell proliferation at 0, 24, 48, and 72 h after transfection. (D) The cell migration ability was determined by the scratch method. (E) Quantiative RT‐PCR detection of *Vimentin* and *α*‐*SMA*. (F) Cell cycle distribution was analyzed by flow cytometry. (G) Cell apoptosis was analyzed by flow cytometry. (H) Western blot was used to detect the expression of CDK2, Cyclin E1, and PCNA. *α‐SMA*, *α‑smooth muscle actin*; CDK2, Cyclin‐dependent kinase 2; PCNA, Proliferating Cell Nuclear Antigen.

Compared with the NC inhibitor group, the results were as follows. After transfection of the miR‐130a‐3p inhibitor into HS68 cells, expression of miR‐130a‐3p in the cells was downregulated (Figure 5A). *MAFB* mRNA expression was upregulated to approximately 2‐fold relative to the control (Figure 5B). The CCK‐8 assay revealed that cell proliferation was promoted on day 2 and day 3 after transfection (Figure 5C). The scratch wound healing assay showed an increase in cell healing rate on day 1 after transfection (Figure 5D); meanwhile, *Vimentin* and *α* *‐SMA* levels were elevated, indicating enhanced cell migration (Figure 5E). Flow cytometry analysis results revealed that the percentage of the G1 stage decreased (Figure 5F) and apoptosis rate decreased (Figure 5G); the expressions of CDK2, CyclinE1, and PCNA were significantly upregulated (Figure 5H), suggesting accelerated cell cycle progression after transfection.

### 3.5 Overexpressing miR‐130a‐3p Inhibits *MAFB* and Cell Functions

**FIGURE 6:**
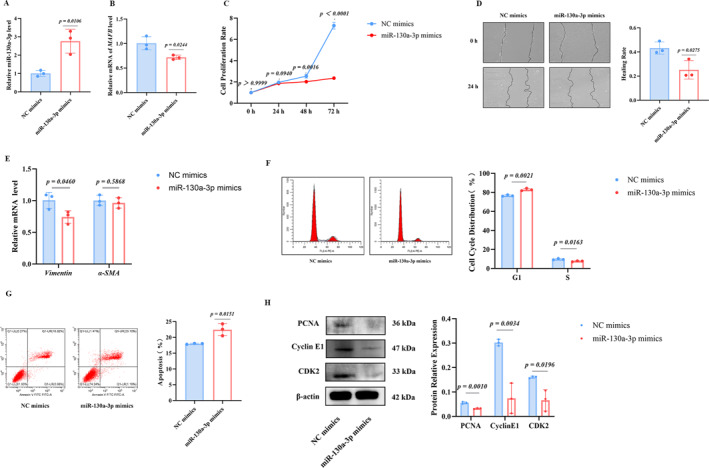
Overexpression of microRNA‐130a‐3p and detection of cell proliferation, cell migration, cell apoptosis, and cell cycle‐related changes in HS68 cells. (A–B) Quantitative RT‐PCR detection of microRNA‐130a‐3p and *MAFB* mRNA. (C) CCK‐8 was used to detect cell proliferation at 0, 24, 48, and 72 h after transfection. (D) The cell migration ability was determined by the scratch method. (E) Quantitative RT‐PCR detection of *Vimentin* and *α*‐*SMA*. (F) Cell cycle distribution was analyzed by flow cytometry. (G) Cell apoptosis was analyzed by flow cytometry. (H) Western blot was used to detect the expression of CDK2, Cyclin E1, and PCNA. *α‐SMA*, *α‑smooth muscle actin*; CDK2, Cyclin‐dependent kinase 2; PCNA, Proliferating Cell Nuclear Antigen.

Compared with the NC mimics group, the results were as follows. After transfection of the miR‐130a‐3p mimics into HS68 cells, miR‐130a‐3p expression in the cells was upregulated (Figure 6A), and *MAFB* mRNA expression was downregulated to 0.7‐fold of the control (Figure 6B). The CCK‐8 assay revealed that cell proliferation was inhibited on day 2 and day 3 after transfection (Figure 6C). The scratch wound healing assay showed a decrease in cell healing rate on day 1 after transfection (Figure 6D); meanwhile, *Vimentin* level was reduced, *α* *‐SMA* level had no significant difference (Figure 6E). Flow cytometry analysis results revealed that the percentage of the G1 stage increased and the percentage of the S stage decreased (Figure 6F) and apoptosis rate increased (Figure 6G); the expressions of CDK2, CyclinE1, and PCNA were significantly downregulated (Figure 6H), suggesting decelerated cell cycle progression, and indicating cell cycle arrest at the G1/S transition after​ transfection.

### 3.6 miR‐130a‐3p Directly Targets *MAFB*

**FIGURE 7:**
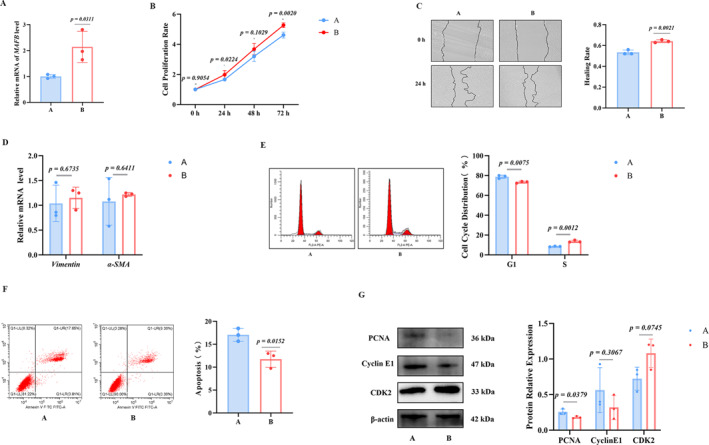
Co‐transfection of microRNA‐130a‐3p mimics and pcDNA3.1‐Mafb and detection of cell proliferation, cell migration, cell apoptosis, and cell cycle‐related changes in HS68 cells. (A) Quantitative RT‐PCR detection of *MAFB* mRNA. (B) CCK‐8 was used to detect cell proliferation at 0, 24, 48, and 72 h after transfection. (C) The cell migration ability was determined by the scratch method. (D) Quantitative RT‐PCR detection of *Vimentin* and *α‐SMA*. (E) Cell cycle distribution was analyzed by flow cytometry. (F) Cell apoptosis was analyzed by flow cytometry. (G)  Western blot was used to detect the expression of CDK2, Cyclin E1, and PCNA. (A.NC mimics + pcDNA3.1‐NC, B.microRNA‐130a‐3p mimics + pcDNA3.1‐*MAFB*). *α‐SMA*, *α‑smooth muscle actin*; CDK2, Cyclin‐dependent kinase 2; PCNA, Proliferating Cell Nuclear Antigen.

In comparison with the group transfected with pcDNA3.1‐NC and NC mimics, the results were as follows. After transfection of the miR‐130a‐3p mimics and pcDNA3.1‐*MAFB* into HS68 cells, *MAFB* mRNA expression in the cells was upregulated to 2‐fold of the control level (Figure 7A). The CCK‐8 assay revealed that cell proliferation was promoted on day 3 after transfection (Figure 7B). The scratch wound healing assay revealed an increase in cell healing rate on day 1 after transfection (Figure 7C); similarly, *V* *imentin* and *α* *‐SMA* showed no significant differences, indicating no notable change in cell migration (Figure 7D). Flow cytometry analysis results revealed that the percentage of the G1 stage decreased and the percentage of the S stage increased (Figure 7E) and apoptosis rate decreased (Figure 7F); the expression of CDK2 and CyclinE1 showed no significant difference, whereas the expression of PCNA showed a significant decrease (Figure 7G). It was miR‐130a‐3p that exerted a targeted regulatory effect on Mafb, as confirmed by the dual‐luciferase reporter gene assay in HEK‐293T cells (Figure 1B).

## 4 Discussion

In this study, we found that miR‐130a‐3p is related to the development of hypospadias. Its expression is higher in the foreskin tissue of children with hypospadias than in that of children in the normal group. A DEHP‐induced hypospadias mouse model was successfully established, and the GT tissue of the model mice also exhibited high miR‐130a‐3p expression in comparison with the normal mice. In cell experiments, miR‐130a‐3p can directly target and regulate Mafb to inhibit proliferation and migration capacity, increase apoptosis, and delay cell cycle progression, participating in the occurrence of hypospadias.

Previous work by our group has demonstrated that *MAFB* expression is lower in foreskin tissues of children with hypospadias than in normal controls and that *Mafb*‐knockout male mice exhibit defective urethral fusion, a phenotype resembling human hypospadias [14, 15], suggesting that the *MAFB* gene is indispensable for the normal development of the urethra, and its abnormal expression may lead to urethral developmental malformations. We verified the results in clinical hypospadias tissues and mouse GT tissues, and performed MAFB‐silenced cell experiments, all results showing that down‐regulation of *MAFB* expression is closely associated with hypospadias‐related abnormal phenotypes, consistent with previous findings.

The occurrence of hypospadias is related to disordered miRNA expression, and miR‐130a‐3p can target androgen receptors, leading to abnormal cell proliferation and migration [13, 16]. Through combined verification at the clinical, animal, and cellular levels, we further supplemented and improved this pathogenesis, confirming that miR‐130a‐3p is not only highly expressed in hypospadias but also can affect cell function by directly targeting *MAFB*, providing a new molecular pathway for miRNA involvement in the pathogenesis of hypospadias.

Epithelial‐mesenchymal transition (EMT) is crucial for the normal morphogenesis of the urethra. As key markers of EMT, abnormal expression of *Vimentin* and *α* *‐SMA* can lead to disordered cell migration function and affect the normal fusion of urethral tissue [17, 18, 19, 20, 21]. In cell experiments, inhibiting miR‐130a‐3p could simultaneously upregulate the expression of *Vimentin* and *α* *‐SMA* and enhance cell migration ability, whereas silencing *MAFB* or overexpressing miR‐130a‐3p only downregulated *Vimentin* expression without a significant effect on *α* *‐SMA*, which may be related to the source and passage number of cells. Cell cycle and cell apoptosis dysregulation also contribute to hypospadias [22, 23]. The CyclinE/CDK2 complex regulates the G1/S transition and initiates DNA replication. Orderly G1‐to‐S progression is essential for normal eukaryotic cell proliferation. Kinase activity of CyclinE/CDK2 peaks during this transition [24, 25]. Furthermore, PCNA expression is closely associated with active cell proliferation [26, 27]. In this study, inhibiting miR‐130a‐3p significantly upregulated the expression of CDK2, Cyclin E1, and PCNA, accelerating the cell cycle process; overexpressing miR‐130a‐3p significantly downregulated the expression of these three molecules, delaying the G1/S phase transition; silencing Mafb downregulated the expression of CyclinE1 and PCNA, further confirming that the miR‐130a‐3p/*MAFB* axis could affect cell proliferation and cycle progression by regulating key molecules such as Cyclin E1, PCNA, and CDK2, participating in the occurrence of hypospadias.

This study has certain limitations and needs further improvement in subsequent studies. Due to the limited sample size, we will expand the sample to verify further. Cell experiments were only conducted in the HS68 cell line under in vitro culture conditions and did not involve other cell types related to urethral development. Although this study successfully established a DEHP‐induced hypospadias mouse model and it is known that *Mafb* gene knockout mice have a similar hypospadias phenotype, the specific expression pattern of miR‐130a‐3p during mouse urethral development, the expression changes at different developmental stages, whether regulating miR‐130a‐3p expression can improve the hypospadias phenotype of the mouse model, and the specific mechanism of their synergistic regulation of urethral fusion in vivo still require further in vivo experimental verification.

## 5 Conclusion

We found that the *MAFB* gene in the occurrence of hypospadias is also influenced by its upstream regulatory factors. Specifically, miR‐130a‐3p can target and inhibit *MAFB* expression, disrupting the normal processes of proliferation, migration, cell apoptosis, and cell cycle progression, and may be involved in the pathogenesis of hypospadias. This study reveals the molecular mechanism by which miR‐130a‐3p targets and regulates *MAFB* in hypospadias from an epigenetic perspective, providing a new theoretical foundation for a deeper understanding of the pathogenic essence of hypospadias.​ It also provides a new insight for clinically preventing the occurrence of hypospadias through miR‐130a‐3p.

## Author Contributions

**Jiaxin Zhou:** conceptualization, methodology, writing – original draft. **Yixuan Wang:** data curation, software. **Zhicheng Zhang:** conceptualization, data curation, software. **Qiang Zhang:** data curation, software. **Zhenmin Liu:** data curation, software. **Hongsong Chen:** investigation, visualization. **Xingguo Luo:** investigation, visualization. **Chunlan Long:** writing – review and editing. **Lianju Shen:** writing – review and editing. **Xing Liu:** writing – review and editing. **Guanghui Wei:** writing – review and editing.

## Funding

We acknowledge the support of the Department of Urology of Children's Hospital of Chongqing Medical University and the financial support of the National Natural Science Foundation of China (Grant 82501925), and the project entitled “Clinical Intervention for Structural Abnormalities of the Pediatric Urogenital System” (Grant CSTB2025TIAD‐LYKJXZJSYYX0005).

## Ethics Statement

This study was approved by the Ethics Committee of the Children's Hospital of Chongqing Medical University (File No. 2024‐141). This study complied with the relevant guidelines and regulations of the Ethics Committee of the Children's Hospital of Chongqing Medical University and was performed in accordance with the Declaration of Helsinki. Written informed consents to participate in this study were provided by the participant's legal guardians/next of kins. All animal experiments were conducted in strict accordance with the ARRIVE guidelines.

## Conflicts of Interest

The authors declare no conflicts of interest.

## Data Availability

Data will be made available on request.
